# Effect of Inclusion of Degraded and Non-Degraded Date Pits in Broilers’ Diet on their Intestinal Microbiota and Growth Performance

**DOI:** 10.3390/ani10112041

**Published:** 2020-11-05

**Authors:** Salem R. Alyileili, Ibrahim E. H. Belal, Ahmed S. Hussein, Khaled A. El-Tarabily

**Affiliations:** 1 Department of Integrative Agriculture, College of Food and Agriculture, United Arab Emirates University, Al-Ain 15551, UAE; ahussein@uaeu.ac.ae; 2 Department of Biology, College of Science, United Arab Emirates University, Al-Ain 15551, UAE; 3 College of Science, Health, Engineering and Education, Department of Agricultural Sciences, Murdoch University, Murdoch, WA 6150, Australia

**Keywords:** broilers, date pits, fungi degraded date pits, growth performance, total bacterial count

## Abstract

**Simple Summary:**

In developing countries, most of the feedstuffs for animal nutrition are imported. Therefore, great attention has been focused on the use of agro-industrial by-products as feedstuffs to improve the feeding value of animal nutrition. These improvements can be induced by different means, including feed additive supplements, such as enzymes, probiotics, prebiotics, and organic acids. Other factors can also induce enhancement such as grinding, autoclaving, pelleting, and solid-state degradation by cellulolytic fungi. These methods aim to enhance the digestion of complex carbohydrates and decrease anti-nutritional constituents. In this study, the impact of non-degraded date pits (NDDP) and degraded date pits (DDP) using the cellulolytic fungus *Trichoderma reesei* in broiler’s diets on the gut bacterial growth and growth performance was investigated. It was found that when DDP are present at a rate of 10% of the broilers’ diet, it boosted gut health by increasing prebiotic production, thus serving as a growth promoter in broilers’ nutrition.

**Abstract:**

The current study aims to assess the effect of non-degraded date pits (NDDP) and degraded date pits (DDP) in broilers’ diets on gut microbiota and growth performance. The degradation of date pits (DP) occurred via the cellulolytic fungus *Trichoderma reesei* by a solid-state degradation procedure. One-day-old Brazilian broilers were allocated into six dietary groups: (1) maize–soy diet, (2) maize–soy diet with oxytetracycline (20%, 50 g 100 kg^−1^), (3) maize–soy diet with 5% NDDP, (4) maize–soy diet with 10% NDDP, (5) maize–soy diet with 5% DDP, and (6) maize–soy diet with 10% DDP. At the end of the trial, the total count of bacteria was significantly (*p* < 0.05) less in broilers fed 10% DDP diet (treatment 6) compared with the control group (treatment 1). In addition, DDP and oxytetracycline control diets have a similar diminishing effect on total bacterial counts and the populations of *Salmonella*, *Campylobacter*, *Shigella* spp., and *Escherichia coli*. Over 35 days of trial, weight gains were similar among the six dietary groups. Our results showed that DDP and control diets have a similar effect on growth performance. The feed conversion ratio (FCR) was poorer in broilers fed NDDP diets than other treatments. The European Production Efficiency Index (EPEI) was greater with 5% and 10% DDP than those fed NDDP at the same levels, with no significant variance from the control and antibiotic-supplemented diet (treatment 2). Overall, it can be suggested that maintaining 10% of DDP can partly replace dietary maize while also serves as a gut health enhancer and thus a growth promoter in the diet for broilers.

## 1. Introduction

Dietary energy is crucial in poultry growth and development. About 60–65% of the poultry diets’ metabolizable energy is derived from sorghum and maize [1]. In the Arabian Gulf countries, raw ingredients for broiler feed are imported, and their price has increased due to antagonism with the food manufacturers and elevated biofuel production [2]. A shortage in global food and feed resources due to the COVID-19 pandemic will result due to competition between humans and animals for cereals and grains. Accordingly, it is most important to enhance the utilization of locally available animal feed ingredients. This is due to the recent global disturbance in food distribution [3]. Thus, the utilization of readily available local energy sources would improve the poultry production in areas with limited feed supply.

Currently, antibiotics are added to poultrys’ diets as growth promoters, yet using them for extended periods lead to antimicrobial resistance (AMR) in the pathogenic bacteria, especially the Gram-negative ones [4]. The use of antibiotics has been banned by the European Union in the feed of food-producing animals [4]. Therefore, there is a need to find alternatives for antibiotics.

Dates from *Phoenix dactylifera* L. palm trees are produced in many arid land areas where grains are not available. The United Arab Emirates (UAE) is a major date-producing country, and the annual production of raw dates reached about 775,000 metric tons in the year 2016 [5]. In date fruit-processing areas, date pits (DP) are usually discarded because they are rich in fibers and contain high levels of nondigestible carbohydrates. The DP is a suitable source of nutrients and contains about 71–103 g kg^−1^, 50–63 g kg^−1^, 99–135 g kg^−1^, 22.5 g kg^−1^, and 10–18 g kg^−1^ of moisture, crude protein, lipid, fiber, and ash, respectively. The total sugars content is 29.3 g kg^−1^ [6]. Additionally, the neutral detergent fiber (NDF) of DP is 650–690 g kg^−1^, and the acid detergent fiber (ADF) of DP is 460–510 g kg^−1^, consisting of total cellulose and lignin [7].

While DP have been currently used as a poultry feed, due to the complex carbohydrates present, there is a limit to their use as a feed ingredient at higher levels [8]. Ground date seed powder could be used as a suitable feed additive to combat aflatoxicosis by evading the adverse impacts of the mycotoxin binders [9].

DP up to 18% is an alternative feedstuff in diets for laying hens with little influence on productive traits, including eggs’ weight [10]. So, DP meals could be added to 25–33-week-old local hen diets with amounts up to 5% without β-mannanase and without affecting growth performance or egg quality [11]. The maize content in broilers’ diets was replaced with DP at an amount of 15% without any adverse impact on carcass percentage and its cuts [12]. Al-Saffar et al. [13] reported that DP can be fed to laying hens up to an amount of 10% DP, rendering them an option for a cheap source of feed with no negative effects. Broilers feed supplemented with DP enhances the immunity antioxidant status in chicken [14]. DP utilization in broilers’ diet at a rate of 4% reduces the deleterious effects of aflatoxin B1 (AFB1) and the feed conversion ratio (FCR) while significantly increasing the relative weight of the liver, intestines, gizzard, and thighs [15].

Nondigestive fibers of DP could partly break down when expose to degrading microbial enzymes such as xylanases, releasing digestible carbohydrates. Solid-state degradation (SSD) is used to improve complex carbohydrates digestion in poultry feed [16]. Additionally, DP degradation by the cellulolytic fungus, *Trichoderma reesei,* was used to improve its digestion by Nile tilapia, *Oreochromis niloticus* [17]. DP fibers are good substrates, cellulose sources, and oligosaccharides for *T. reesei* growth and activation using their catabolic enzymes such as cellulases, hemicellulases, and pectinases [18]. Simple carbohydrate molecules can be produced from DP fiber with the help of exogenous degrading microbial enzymes such as xylanases, and they may be better utilized by animals.

There is a limited amount of research focusing on degraded date pits (DDP) and its utilization as poultry feed, even though they contain a potentially useful quantity of nutrients essential for poultry health. Therefore, this study investigated the effect of inclusion of DDP in broilers’ diet on their gut microbiota and growth performance.

## 2. Materials and Methods

This trial was approved, under protocol number (313072), by the College of Food and Agriculture, United Arab Emirates University, Al-Ain, United Arab Emirates. The university recommends maintaining animal welfare, inducing minimal stress and possible maximum rights, and avoiding any harm or suffering to the animals under research in agreement with the International Guidelines for Research Involving Animals (Directive2010/63/EU).

### 2.1. Date Pits

The DP (*P. dactylifera* L.) (Raziz variety) were collected from a date factory in Al-Ain city, UAE. A medium-size grinder was used (Skiold, Saeby, Denmark) to produce one mm diameter DP.

### 2.2. T. reesei Culture Preparation

*T. reesei* was applied as described by Hussein et al. (2017) [19]. Lyophilized *T. reesei* ampoules (Deutsche Sammlung von Mikroorganismen und Zellkulturen GmbH, (DSMZ), Braunschweig, Germany) were used to produce rehydrated *T. reesei* culture; then, it was inoculated to a potato dextrose broth (PDB; Lab M Ltd., Lancashire, UK) modified with 250 µg mL^–1^ chloramphenicol (Sigma–Aldrich Chemie GmbH, Taufkirchen, Germany) and 100 µg mL^–1^ streptomycin sulfate (Sigma–Aldrich, Taufkirchen, Germany). Using a rotary shaker (Model G76, New Brunswick Scientific, Edison, NJ, USA) the flasks were incubated at 250 rpm at 25 ± 2 °C for seven days in the dark to produce the subsample. Fungal growth in the flasks was examined daily.

### 2.3. Preparation of DDP

Using the SSD, DDP were prepared within an incubator as described by Hussein et al. [19]. DP substrate was sterilized at 121 °C for 30 min, and then it was added to the system of SSD. *T.* *reesei* subsamples were supplemented to the SSD system and were spread to every cone. Then, the system units were covered to prevent contamination with constant moistened air that was disinfected with ultraviolet rays; then, they were integrated within the system, which was preserved at 90% humidity in a dark room at 30 ± 2 °C for three weeks [19]. Then, DDP was collected and kept at a 4 °C temperature until use [19].

### 2.4. Starter and Finisher Diets

Six isonitrogenous and isocaloric diets were formulated for starter and finisher diets separately. The six dietary treatments were established as, T1: control, maize–soy diet; T2: maize–soy diet + antibiotic (oxytetracycline 20%, 50 g 100 kg^−1^); T3: maize–soy diet with 5% non-degraded date pits (NDDP); T4: maize–soy diet with 10% NDDP; T5: maize–soy diet with 5% DDP; and T6: maize–soy diet with 10% DDP. The levels of DP were chosen based on previous works [13,14,15] considering the level of undigested carbohydrates and the available amount of DP.

The nutrient composition of the experimental diets was calculated using the method provided by Hashim et al. [20]. Feed ingredients were finely ground separately; then, they were weighed and mixed using a Spar mixer, 3HP, Taiwan for 25 min. The prepared diets are shown in Table 1 and Table 2.

### 2.5. Birds and Dietary Treatments

The study was conducted as per the guidelines of the UAE University Ethical Committee Guidelines for animal research and care. Males were used herein rather than mixed sex to achieve homogeneity in the variance within replicates and to eliminate experimental error. In addition, males are also fast-growing animals rather than females and thus can express the treatments’ variance better than using mixed sex and/or females. This was done to improve the precision of analyses of variance.

For performance traits, a total of 198, one-day-old Brazilian male broiler chicks “Cobb 500” were randomly divided into six dietary treatments in a straight run experimental design (one-way analyses of variance) of three replicates of 11 chicks each, and the replicate was the experimental unit. In the microbiological study described below in Section 2.6, the number of samples used was three samples (replicates) per treatment considering the sample (replicate) as an experimental unit. The samples collected represented all treatment replicates. Chickens were housed in separate brooding battery cages (50 × 45 × 45 cm) as 11 chicks per cage in an environmentally controlled house. Broilers were fed and watered ad libitum level for 35 days. The experimental diets were fed within the starter and finished period (from 1 up to 21 days and from 22 up to 35 days, respectively). The rearing and feeding practices were similar to those previously cited [21,22].

### 2.6. Enumeration of Bacterial Populations

On the last day of the experiment, chickens in every cage were weighed to get the average weight for each group. This was followed by randomly selecting three chickens from each group to be slaughtered for gut sample collection.

The total bacterial count and the population of some selected bacteria comprising of *Salmonella* spp., *Campylobacter* spp., *Shigella* spp., and *Escherichia coli* within the guts of chickens’ samples were determined using the tenfold dilution plate technique [23]. The chicken gut (three per treatment) was firstly washed seven times with sterilized deionized water for 15 min each to eliminate surface microorganisms, and the gut fresh weight was recorded before any additional processing. To prevent the diffusion of the commercial bleach, which was used as a surface-sterilizing material inside the guts and to equilibrate the gut osmotic pressure, the washed guts were immersed into a phosphate-buffered saline solution (PBS) (pH 7.0) for ten minutes [24]. The PBS was filter sterilized using a 0.22 μm filter-sterilized membrane (Millipore Corporation, Burlington, MA, USA). The surface-sterilized chicken gut became so by firstly soaking the gut in a 70% ethanol solution for five minutes. This was followed by a further surface-sterilization in 1% sodium hypochlorite solution (Clorox) for one more minute. Then, the guts were washed seven times in PBS where each rinse was three minutes long [25]. Then, the samples were rinsed ten times with sterilized deionized water. Tween 20 (Sigma–Aldrich) (0.05 mL^−1^) was used in all the surface sterilization procedures using Clorox. Chicken guts were blended in 100 mL of PBS using a sterilized tissue homogenizer (OCI Instruments, Omni Corporation International, Waterbury, CT, USA) under aseptic conditions. Then, the gut macerate (triturate) was shaken on a rotary shaker (Model G76, New Brunswick Scientific, Edison, NJ, USA) for one hour at 250 rpm and 25 °C. Then, the macerate suspension was further placed in an ultrasonicator for 55,000 cycles’ s^−1^ for 20 s (Biologics, Inc., Manassas, VA, USA). The macerate suspension was filtered via a sterilized No. 1 Whatman filter paper (Whatman, Maidstone, England); the filtrate was diluted in serials (10^−2^–10^−5^) in sterilized PBS [24]. From these dilutions, 0.2 mL were spread across the surface of various common purposes and selective agar media in sterilized plastic petri dishes (90 mm diameter) using sterilized L-shaped spreaders. All air-dried plates became so in a laminar flow-cabinet, where they were dried for 15 min and incubated in a dark incubator for four days at 37 °C. For each dilution, five plates were allocated for each sample. The groups were selected as follows: (1) total bacteria on nutrient agar medium (Lab M Ltd., Heywood, Lancashire, UK), (2) *Salmonella* spp. on Bismuth sulfite agar (modified Wilson and Blair medium (CM0201) (Oxoid Ltd., Basingstoke, Hampshire, UK), (3) *Campylobacter* spp. on Campy (Cefoperazone, Vancomycin, and Amphotericin B) agar medium (Hardy Diagnostics, Santa Maria, CA, USA), (4) *Shigella* spp. on xylose lysine deoxycholate agar (XLD agar M031) (HiMedia Laboratories Ltd., Mumbai, India), and (5) *Escherichia coli* on Eosin Methylene Blue (EMB) (M317) (HiMedia). The bacterial colony counts were done at the termination of the incubation period (four days), and the total bacterial population was expressed as log_10_ colony-forming units (cfu) g fresh gut tissue^−1^.

### 2.7. Growth Performance

Feed intake (FI) was measured weekly. Growth performance, including FI, body weight gain (BWG), and FCR, were assessed. During the experimental period, mortality was recorded daily, and mortality rates were calculated accordingly in each replicate. The European Production Efficiency Index (EPEI) was deduced as previously reported [21,22].

### 2.8. Statistical Analysis

Statistical analysis was carried out using the statistical package for social sciences (SPSS Version 20.0 for windows: SPSS Inc., Chicago, IL, USA). Analysis of variance (ANOVA) was applied to study the effect of collected data concerning the effects of DDP and NDDP amendment on total and specific bacterial populations. The data for microbiological and growth performance tests were subjected to analysis of variance (ANOVA). The experimental unit was the replicate and the model was:

Yij = μ + αi + εij,

where Yij is the observed value of the individual treatment i, μ is the overall mean, αi is the fixed deviation of the mean of group i from the grand mean μ, and εij is the random error effect. The same model was used for microbiological traits using three samples per treatment, and the sample was the experimental unit. Fisher’s protected least significant difference (LSD) test at *p* = 0.05 was applied to compare significant differences among means for all analyses. Before analysis of variance, all percentage data were transformed to their analogous arcsine.

## 3. Results

### 3.1. Enumeration of Bacterial Populations

The gut samples of broilers without DDP and NDDP (treatment 1, control) had a considerably (*p* < 0.05) greater pathogenic bacterial counts (Table 3). A substantial diminishing (*p* < 0.05) in bacterial populations was evident in the gut samples with DDP and NDDP compared with the samples without DP (control, treatment 1). The appraised total populations of total aerobic bacteria, *Salmonella* spp., *Campylobacter* spp., *Shigella* spp., and *Escherichia coli* were considerably (*p* < 0.05) lesser in the gut samples of broilers consuming DDP and NDDP (treatments 3–6) compared to the gut tissues of broilers fed without DP (control, treatment 1) (Table 3). A marked decline (*p* < 0.05) in populations of bacteria was evident by elevating the DDP levels. The groups fed 10% DDP (treatment 6) significantly (*p* < 0.05) surpassed other treatments in quashing the total bacterial counts and also showed a reduced total number of *Salmonella* spp., *Campylobacter* spp., *Shigella* spp., and *Escherichia coli*. This particular treatment was nearly as good as (*p* > 0.05) the antibiotic treatment, which included the addition of oxytetracycline (treatment 2) (Table 3).

### 3.2. Effects of NDDP and DDP on the Average BWG and Growth Parameters

The effects of NDDP and DDP on the average BWG of broilers are shown in Table 4. The overall BWG in broilers fed through all the experimental diets was not significantly different (*p* > 0.05) during either the starter or the total feeding periods (Table 4).

FI during both the starter and total periods were similar (*p* > *0.05*) in broilers fed the control, control with the antibiotic oxytetracycline, 5% and 10% DDP diets, while they were markedly lower (*p* < 0.05) than those fed 5% and 10% NDDP diets (Table 4).

The FCR during the starter and entire periods were similar (*p* > 0.05) among broilers fed all test diets. Additionally, the survival rate was similar among all tested groups (Table 4).

The EPEI was higher in groups fed DDP and control with or without the antibiotic oxytetracycline than those fed NDDP. In addition, the differences emerged between NDDP concentrations: the index was the lowest in the groups fed 5 and 10% NDDP (Table 4).

## 4. Discussion

Feed quality, dietary nutrients composition, and profiles impacted the health and growth performance of animals. Oat, wheat, barley, and maize are now largely fed as constituents in poultry diet [1]. Natural carbohydrate polymers have prebiotic property, and nowadays, they are widely used in poultry feeds. DDP by the fungus *T. reesei* is a good source of carbohydrates and fibers [17,26], which can be used as poultry feed.

The present results indicate that the populations of total bacteria including pathogenic strains of *Salmonella* spp., *Campylobacter* spp., *Shigella* spp., and *Escherichia coli* were different based on diet composition and the fungal degrading process. A balanced microbial population in the intestinal tract is vital to growth, and these microbes maintain the health of the birds [21]. Bacteria present in the intestine metabolize the nutrients present in the diets by synthesizing some vitamins, lactic acid, and short-chain fatty acids [27]. In the literature, microbial populations in gut vary with animal species and their interaction with host animals depending on age, dietary constituents, and the environment of gastrointestinal tract [13,28,29,30]. Some plant substitutes and additives have probiotic and/or prebiotic effects on gut microflora in direct and indirect ways [30,31,32]. Research indicates that plant-based nutraceuticals enhanced broilers’ productivity as well as antibiotic growth promoters [33,34]. Immune enhancement due to the activity of antimicrobial aspects immensely inspired mechanisms through which phytogenic substances and dietary modification exert beneficial properties on the wellbeing and performance of birds under normal and stress conditions [35,36,37,38]. In birds, the nutritional advantage received from microflora pales in comparison with other animal species, as most occur in the hind gut [21]. Broilers fed with 5% and 10% DDP diet in the current study showed a significant reduction in the total bacterial count in their gut in which a 10% DDP diet was most effective when compared with other dietary treatments. Similarly, broilers fed with 5% and 10% DDP diet also showed significant reduction in the population of pathogenic bacteria such as *Salmonella* spp., *Campylobacter* spp., *Shigella* spp., and *Escherichia coli* compared with other dietary treatments with the 10% DDP diet being the most effective. Other studies reported that herbal seeds were principally concomitant with the lowered number of *Bacteroides* spp., *Enterococcus* spp., and *Escherichia coli* considering the antibiotic treatment and the control groups [39,40,41].

In the present study, the results indicated that DP inclusion in broilers’ diets has a similar effect on BWG as dietary maize. Vandepopuliere et al. [27] found that the broiler chicks fed with NDDP gave the same growth performance as the control group. In addition, a significant improvement in weight gain was found in broilers when fed a diet with DP at levels of 5%, 10%, and 15% and showed similar body weight when compared to antibiotic diet-fed chicks [42,43,44]. The partial replacement of corn in broiler diets by 15% of NDDP showed no marked effects on growth and FI during the starter–grower period up to week 5 of age [12]. Recent research showed that NDDP between 2% and 10% have significant effects on broiler health and induced higher body weight [14,44]. All these data are establishing evidence that using NDDP to feed broiler chickens supports their growth and improves the average weight gain, even though DP do not have the same amount of digested carbohydrates when compared with other cereals such as maize, wheat, oats, and barley. That was explained by the presence of natural anabolic substances in DP, which may boost the growth rate and feed utilization efficiency of animals [45]. Additionally, DP contain growth promoters such as galactomannan and mannose [13,21].

Our results showed that broiler diets including NDDP or DDP did not induce much of a difference in the BWG of broilers when compared with those fed corn diets. However, FI was higher and the FCR was impaired in broilers fed the 5% or 10% NDDP diets as compared with DDP treatments. This indicates that the degradation of DP improved FI and FCR. Belal [17] explained the improvement in FCR of DP after degradation with *T. reesei* as a result of partly breaking the nondigestible fibers to digestible carbohydrates. Juanpere et al. [46] indicated that xylanases and β-glucanases in wheat and barley diets reduced the viscosity of the digesta by 30–50% due to digestible carbohydrates. A viscosity reduction leads to improved digestibility, apparent metabolizable energy, feed consumption, BWG, and FCR. The digestible carbohydrates in DP resulted in improved growth performance by increasing the digestibility and gut microbiota. The present findings agree with those of Jumah et al. [47] and El-Faham et al. [48], who indicated that the FI of chicks receiving NDDP in their diet was better than those fed the control diet. DP provides a cheap source of feed, and using 10% NDDP had no adverse effects on the performance of the chickens [49,50]. The present results indicate that DDP at 10% had no harmful effect on FCR and EPEI, and similar results were previously reported [13,51]. Overall, the results in the current study indicate a possible replacement for 10% of the maize–soybean by DDP in broiler feed.

The implementation of agricultural wastes such as DP in poultry feed [52,53,54,55] will currently play an important role in replacing the expensive feed [3]. In the current study, the application of DDP in poultry feed will be of vital importance under the circumstances of COVID-19 due to the expected worldwide shortage in animal feed supply and the expected damages in the agriculture sector [3]. It could be suggested that further experiments are needed to support the present findings and to evaluate the interest of the use of DP for the feeding of broilers, and if the addition of the fungus *T. reesei* would affect the performance of the animals.

## 5. Conclusions

The cellulolytic enzymes of *T. reesei* were successful in degrading DP and producing positive structural changes in the DP and thus improving gut health through decreasing the total bacterial counts and the populations of harmful microbiota including *Salmonella* spp., *Campylobacter* spp., *Shigella* spp., and *Escherichia coli.* The enzymes produced by *T. reesei* have reduced the complex carbohydrates to more simple and beneficial ones. It was concluded that NDDP and DDP could be used to partly replace dietary corn and additionally, the inclusion of 10% DDP in the broilers’ diet (treatment 6) resulted in better broilers’ gut health without the use of antibiotics.

## Figures and Tables

**Table 1:** The composition of the six experimental starter test diets and their calculated nutrients composition (starter period from day 1 to day 21).

Ingredients in g kg^−1^	Test Diets					
	T1	T2	T3	T4	T5	T6
Maize	594.0	594.0	537.0	466.0	537.0	466.0
Soybean meal	320.0	320.0	307.0	312.0	307.0	312.0
Sodium chloride	4.0	4.0	3.8	3.8	3.8	3.8
Limestone	11.0	11.0	10.5	11.0	10.5	11.0
Dicalcium phosphate	15.6	15.6	12.2	12.0	12.2	12.0
Vit. + Min. Premix *	10.0	10.0	10.0	10.0	10.0	10.0
DL-Methionine	2.4	2.4	2.4	2.5	2.4	2.5
Lysine	-	-	1.0	1.0	1.0	1.0
Corn oil	20.0	20.0	33.5	50.2	33.5	50.2
Fish meal	23.0	23.0	32.0	31.0	32.0	31.0
Oxytetracycline	-	0.5	-	-	-	-
Non-degraded date pits	-	-	50.0	100.0	-	-
Degraded date pits	-	-	-	-	50.0	100.0
Calculated Nutrients Composition						
Protein (g)	220.0	220.0	219.2	216.2	216.1	216.1
Metabolizable energy, MJ/kg diet	12.55	12.55	12.34	12.34	12.34	12.34
Methionine (g)	5.4	5.4	5.9	5.9	5.9	5.9
Methionine + cysteine (g)	9.1	9.1	9.6	9.4	9.4	9.6
Lysine (g)	12.2	12.2	12.2	12.2	12.4	12.2
Calcium (g)	10.0	10.0	10.0	10.0	10.0	10.0
Available phosphorus (g)	4.6	4.5	4.6	4.5	4.5	4.6

Test diets, T1: control, maize–soy diet; T2: maize–soy diet + antibiotic (oxytetracycline 20%, 50 g 100 kg^−1^); T3: maize–soy diet with 5% non-degraded date pits; T4: maize–soy diet with 10% non-degraded date pits; T5: maize–soy diet with 5% degraded date pits; and T6: maize–soy diet with 10% degraded date pits. * The following was provided per kilogram of diet: vitamin A, 8820 IU; vitamin D3, 2822 IU; vitamin E, 26 IU; menadione dimethyl pyrimidinol bisulfite, 2.0 mg; thiamine, 5.94 mg; riboflavin, 6.2 mg; pantothenic acid, 15 mg; niacin, 44 mg; pyridoxine, 4.5 mg; biotin, 0.23 mg; choline, 1.45 mg; folacin, 0.88 mg; vitamin B12, 0.14 mg; ethoxyquin, 125 mg; selenium, 0.24 mg; copper, 8 mg; iodine, 1.5 mg; iron, 120 mg; manganese, 83 mg; zinc, 60 mg; and cobalt, 5 mg.

**Table 2:** The composition of the six experimental finisher test diets and their calculated nutrients composition (finisher period from day 21 to day 35).

Ingredient in g kg^−1^	Test Diets					
	T1	T2	T3	T4	T5	T6
Maize	646.0	646.0	582.5	521.4	582.5	521.4
Soybean meal	284.0	284.0	277.2	260.0	277.2	260.0
Sodium chloride	4.2	4.2	3.6	3.3	3.6	3.3
Limestone	13.3	13.3	12.2	11.5	12.2	11.5
Dicalcium phosphate	10.5	10.5	9.5	8.0	9.5	8.0
Vit. + Min. Premix *	2.0	2.0	2.0	2.0	2.0	2.0
DL-Methionine	2.0	2.0	2.0	3.0	2.0	3.0
Lysine	1.0	1.0	1.0	1.8	1.0	1.8
Corn oil	25.0	25.0	4.05	5.9	4.05	5.9
Fish meal	1.2	1.2	1.95	3.0	1.95	3.0
Oxytetracycline	-	0.5	-	-	-	-
Non-degraded date pits	-	-	50.0	100.0	-	-
Degraded date pits	-	-	-	-	50.0	100.0
Calculated Nutrients Composition						
Protein (g)	200.0	200.0	199.5	199.7	199.7	200.0
Metabolizable energy, MJ/kg diet	13.34	13.34	12.84	12.59	12.59	13.05
Methionine (g)	5.1	5.1	5.6	5.6	5.6	5.6
Methionine + cysteine (g)	8.5	8.5	9.0	8.9	8.9	9.0
Lysine (g)	10.7	10.7	10.9	11.0	11.0	10.9
Calcium (g)	9.0	9.0	9.0	9.0	9.0	9.0
Available phosphorus (g)	3.6	3.6	3.6	3.6	3.6	3.6

Test diets, T1: control, maize–soy diet; T2: maize–soy diet + antibiotic (oxytetracycline 20%, 50 g 100 kg^−1^); T3: maize–soy diet with 5% non-degraded date pits; T4: maize–soy diet with 10% non-degraded date pits; T5: maize–soy diet with 5% degraded date pits; and T6: maize–soy diet with 10% degraded date pits. * The following was provided per kilogram of diet: vitamin A 8820 IU; vitamin D3, 2822 IU; vitamin E, 26 IU, menadione dimethylpyrimidinol bisulfite, 2.0 mg; thiamin, 5.94 mg; riboflavin, 6.2 mg; pantothenic acid, 15 mg; niacin, 44 mg; pyridoxine, 4.5 mg; biotin, 0.23 mg; choline, 1.45 mg; folacin, 0.88 mg; vitamin B12, 0.14 mg; ethoxyquin, 125 mg; selenium, 0.24 mg; copper, 8 mg; iodine, 1.5 mg; iron, 120 mg; manganese, 83 mg; zinc, 60 mg; and cobalt, 5 mg.

**Table 3:** Bacterial population densities in gut of broiler chickens fed different levels of non-degraded and degraded date pits.

Test Diets	Population Density (log_10_ Colony-Forming Units g Fresh Gut Tissue^−1^)				
	Total Bacterial Counts	*Salmonella* spp.	*Campylobacter* spp.	*Shigella* spp.	*Escherichia coli*
T1	8.53 ^a^	2.03 ^a^	2.74 ^a^	2.12 ^a^	7.19 ^a^
T2	4.21 ^e^	0.031 ^d^	0.081 ^d^	0.531 ^de^	2.06 ^e^
T3	7.07 ^b^	1.41 ^b^	2.01 ^b^	1.90 ^ab^	5.53 ^b^
T4	6.02 ^c^	1.48 ^b^	0.95 ^c^	1.67 ^b^	4.61 ^c^
T5	4.99 ^d^	0.611 ^c^	0.10 ^d^	0.781 ^c^	3.06 ^d^
T6	3.95 ^e^	0.051 ^d^	0.061 ^d^	0.251 ^d^	2.21 ^e^
Pooled SEM	0.381	0.083	0.068	0.082	0.153
*p* value	*	**	*	*	**

Means within a column with different superscripts are significantly different (*p* < 0.05); SEM, standard error of mean; *p* value, probability level; * *p* value of 0.05; ** *p* value of 0.01. Test diets, T1: control, maize–soy diet; T2: maize–soy diet + antibiotic (oxytetracycline 20%, 50 g 100 kg^−1^); T3: maize–soy diet with 5% non-degraded date pits; T4: maize–soy diet with 10% non-degraded date pits; T5: maize–soy diet with 5% degraded date pits; and T6: maize–soy diet with 10% degraded date pits.

**Table 4:** Body weight gain, feed intake, and feed conversion ratio of broiler chickens (g/bird) fed different levels of non-degraded and degraded date pits.

Test Diets	BWGSP (g)	BWGEP (g)	FISP (g)	FIEP (g)	FCRSP	FCREP	Survival Rate (%)	EPEI
T1	866 ^a^	1694 ^a^	1201 ^b^	2421 ^b^	1.39 ^b^	1.43 ^b^	100 ^a^	282 ^ab^
T2	830 ^a^	1729 ^a^	1168 ^b^	2450 ^b^	1.42 ^b^	1.42 ^b^	100 ^a^	291 ^a^
T3	923 ^a^	1779 ^a^	1473 ^a^	2739 ^a^	1.60 ^a^	1.54 ^a^	100 ^a^	275 ^b^
T4	900 ^a^	1730 ^a^	1375 ^a^	2690 ^a^	1.54 ^a^	1.56 ^a^	100 ^a^	264 ^c^
T5	836 ^a^	1719 ^a^	1200 ^b^	2438 ^b^	1.44 ^b^	1.42 ^b^	100 ^a^	288 ^a^
T6	840 ^a^	1753 ^a^	1215 ^b^	2542 ^b^	1.45 ^b^	1.45 ^b^	100 ^a^	288 ^a^
Pooled SEM	34.0	37.4	32.5	0.44	0.038	0.038	0.00	7.3
*p* value	ns	ns	*	*	*	**	ns	*

Means within a column with different superscripts are significantly different (*p* < 0.05); SEM, standard error of mean; *p* value, probability level; * *p* value of 0.05; ** *p* value of 0.01; ns, not significantly different. Test diets, T1: control, maize–soy diet; T2: maize–soy diet + antibiotic (oxytetracycline 20%, 50 g 100 kg^-1^); T3: maize–soy diet with 5% non-degraded date pits; T4: maize–soy diet with 10% non-degraded date pits; T5: maize–soy diet with 5% degraded date pits; and T6: maize–soy diet with 10% degraded date pits. BWGSP, Body Weight Gain Starter Period; BWGEP, Body Weight Gain Entire Period; FISP, Feed Intake Starter Period; FIEP, Feed Intake Entire Period; FCRSP, Feed Conversion Ratio Starter Period; FCREP, Feed Conversion Ratio Entire Period; EPEI, European Production Efficiency Index.
